# Tendon Extracellular Matrix Promotes Myotendinous Junction Protein Expression in Engineered Muscle Tissue under Both Static and Mechanically Stimulated Culture Conditions

**DOI:** 10.1155/2023/6658543

**Published:** 2023-08-29

**Authors:** Lewis S. Gaffney, Matthew B. Fisher, Donald O. Freytes

**Affiliations:** ^1^Joint Department of Biomedical Engineering, North Carolina State University, University of North Carolina at Chapel Hill, Raleigh, NC 27695, USA; ^2^Department of Orthopaedics, University of North Carolina School of Medicine, Chapel Hill, NC 25799, USA

## Abstract

Studying the crosstalk between the muscle and tendon tissue is an important yet understudied area in musculoskeletal research. *In vitro* models can help elucidate the function and repair of the myotendinous junction (MTJ) under static and dynamic culture conditions using engineered muscle tissues. The goal of this study was to culture engineered muscle tissues in a novel bioreactor in both static and mechanically stimulated cultures and evaluate the expression of MTJ-specific proteins within the muscle-tendon unit(paxillin and type XXII collagen). C2C12 myoblasts were seeded in hydrogels made from type I collagen ortendon-derived extracellular matrix (tECM) and allowed to form around movable anchors. Engineered tissues were allowed to form and stabilize for 10 days. After 10 days in the culture, stimulated cultures were cyclically stimulated for 3 hours per day for 2 and 4 weeks alongside static cultures. Strain values at the maximum displacement of the anchors averaged about 0.10, a target that has been shown to induce myogenic phenotype in C2C12s. Protein expression of paxillin after 2 weeks did not differ between hydrogel materials in static cultures but increased by 62% in tECM when mechanically stimulated. These differences continued after 4 weeks, with 31% and 57% increases in tECM tissues relative to type I collagen. Expression of type XXII collagen was similarly influenced by hydrogel material and culture conditions. Overall, this research combined a relevant microenvironment to study muscle and tendon interactions with a novel bioreactor to apply mechanical strain, an important regulator of the formation and maintenance of the native MTJ.

## 1. Introduction

Injuries and disorders involving muscle and tendon tissue are of significant interest. Still, most research investigating these tissues is limited to one tissue type, despite their function as a muscle-tendon unit. There is a relative lack of understanding regarding how the crosstalk between these tissues improves or hinders the healing of the muscle-tendon unit as a whole. To study how the interplay between the muscle and the tendon affects development and healing, *in vitro* models offer a unique opportunity for studying crosstalk among cells in a tissue-specific manner and under relevant microenvironments. Specifically, the myotendinous junction (MTJ) is of interest due to its specialized function within the muscle-tendon unit.

The integrin-mediated complex is localized to the MTJ and anchors to tendon extracellular matrix (ECM) through *α*1*β*7 integrins [1]. Paxillin, vinculin, tensin, and talin are intermediate proteins that anchor the f-actin of the sarcomere to *α*1*β*7 integrins. In response to loading, integrin-mediated complexes increase, forming more focal adhesions to the tendon matrix [2, 3]. In addition, ECM proteins such as type XXII collagen have been isolated at the MTJ with proteomic analysis [4, 5]. Type XXII collagen is a basement membrane protein localized to tissue interfaces, especially the myotendinous junction [6, 7]. Type XXII collagen is thought to support mechanical stability at the MTJ through interactions with *α*1*β*7 integrins [6, 8]. Within the muscle-tendon unit, these proteins are localized in high amounts, making them targets for modeling the MTJ.

Current methods used to study muscle and tendon units *in vitro* have implemented different fabrication techniques such as biphasic electrospun materials [9], self-assembling cell constructs with different cell types [10], 3D printing techniques to organize muscle and tendon cells [11–13], and decellularized muscle-tendon units [14]. In addition, our previously reported model using tissue-specific ECM hydrogels provided a more relevant microenvironment and more relevant cell-matrix interactions than previous methods [15]. Tendon ECM is a useful hydrogel to mimic the native tissue to which muscle cells anchor. Specific interactions within this *in vitro* system, i.e., myoblasts interacting with tendon ECM, can be controlled. In this system, paxillin and type XXII collagen protein expression (MTJ-specific proteins) increased in tendon ECM hydrogels compared to type I collagen hydrogels.

In addition to the interaction of cells and their microenvironment around the MTJ, it is known that mechanical stimulation plays a significant role in the development and healing of the muscle-tendon unit [16]. During tendon development and healing, mechanical stimulation is critical for developing the hierarchal structure and function found in tendon tissue [17–19]. Normal muscle tissue has a high tissue turnover and regeneration capacity in response to mechanical loading [3, 20, 21]. The MTJ is also heavily influenced by mechanical loading. For example, gene expression of talin, a protein involved in the integrin-associated complex, is regulated by mechanical loading [22]. In addition, paxillin and integrin adhesions at the end of muscle fibers accumulate and increase in response to mechanical loading [23, 24].

*In vitro* models of musculoskeletal tissues often involve bioreactors to provide cyclic or static mechanical stimuli to tissue constructs, including tendon constructs [25–29]. For modeling muscle tissue, C2C12 mouse myoblasts are an established cell line to study mechanotransduction in muscle tissue using materials such as fibrin, type I collagen, and decellularized ECM [30–34]. These studies demonstrated that C2C12s cultured under mechanical stimuli exhibit characteristics of mature muscle cells. Overall, there are advantages to modeling these tissue types in mechanically active environments, which improve the tissue-specific activity within the engineered muscle tissue constructs. Interestingly, limited *in vitro* models explore how mechanical stimulation influences the interface where muscle cells attach to the tendon matrix.

During healing, loading can be beneficial or detrimental to tissue function after recovery [19, 20]. Studying mechanisms that occur after overuse in healthy and diseased tissue of the muscle-tendon unit could offer insight to alleviate the loss of function following such injuries or in cases of muscle-tendon disorders. Muscle-tendon *in vitro* models that could recapitulate mechanical loading at the MTJ would be useful in studying homeostasis of the junction and the muscle-tendon unit as a whole, as well as being an important consideration for future incorporation of other tissue types. Tendon-specific ECM was identified as a hydrogel material that could produce MTJ-like phenotypes in C2C12 myoblast cells [15]. These tissue constructs facilitate studying potential cell-matrix interactions that can promote muscle cell anchoring to the tendon matrix. The current work aims to study the effect of cyclic mechanical strain applied on engineered tissue constructs with myoblast cells encapsulated in tendon ECM. Cyclic loading in tendon ECM tissues was compared to tissues without loading and tissues made from type I collagen cultured in loading and nonloading conditions. Overall, this research expands the application of tissue-specific ECM to study the matrix and cell interactions and how mechanotransduction plays a role in the formation of the myotendinous junction.

## 2. Methods

### 2.1. C2C12 Cell Culture for Tissue-Engineered Constructs

Mouse myoblast cells, C2C12s (ATCC), were cultured in regular growth media consisting of Dulbecco's modified Eagle medium (DMEM) (Gibco), 10% v/v fetal bovine serum (Gibco), 1% v/v penicillin/streptomycin (Gibco), and 1% v/v sodium pyruvate (Gibco). The cultured cells were expanded with medium changes every 2-3 days and used between passages 5 and 8. The cells were passaged with 0.25% trypsin/EDTA in HBSS (Gibco). The cells were passaged and resuspended at a density of 6 × 10^6^ cells/mL for seeding in the cell-laden hydrogels.

### 2.2. Tendon-Derived ECM

Tendon ECM (tECM) was derived from porcine Achilles tendons from adult pigs (NCSU Swine Education Unit, Raleigh, NC) after euthanasia for other IACUC-approved studies. Tendon tissues were washed and frozen at −80°C. The tissues were decellularized with adaptations from a previous method [15]. Briefly, the Achilles tendons were washed in 50 mL conical tubes with the following protocol: 0.2% trypsin/EDTA at 37°C for 2 hr, deionized water (DI water) for 30 min, 2X DPBS for 30 min, 2% sodium deoxycholate for 5 hr, DI water for 30 min, 2X DPBS for 30 min, 2% sodium deoxycholate for 14–16 hr, 1% Tween 20 for 1 hr, DI water for 30 min, 0.1% peracetic acid/4% ethanol for 2 hr, 1X DPBS for 30 min (repeated a second time), and DI water for 30 min (repeated a second time). Previously, these methods successfully decellularized muscle and tendon tissue, confirmed with DNA quantification and histology [15]. Decellularized tissues were then lyophilized and ground in a mill to yield a powder, which was stored for up to a year before use.

Tissue-specific hydrogels from the decellularized tendons were produced from the powder using previously described methods [35]. ECM powder was digested with a 20 : 1 weight ratio of pepsin from porcine submucosa (Sigma-Aldrich) in 0.01 M HCl at a concentration of 10 mg/mL. After 48 hours or complete digestion, tissue-specific digests were stored for later use at 4°C. Self-assembling hydrogels were made by using 600 *μ*L of the ECM digest, 60 *μ*L of 0.1 M NaOH, 67 *μ*L of 10X DPBS, and 275 *μ*L of 1X DPBS for a final concentration of 6 mg/mL of tendon ECM hydrogels (tECM).

### 2.3. Bioreactor Fabrication and Assembly

**Figure 1:**
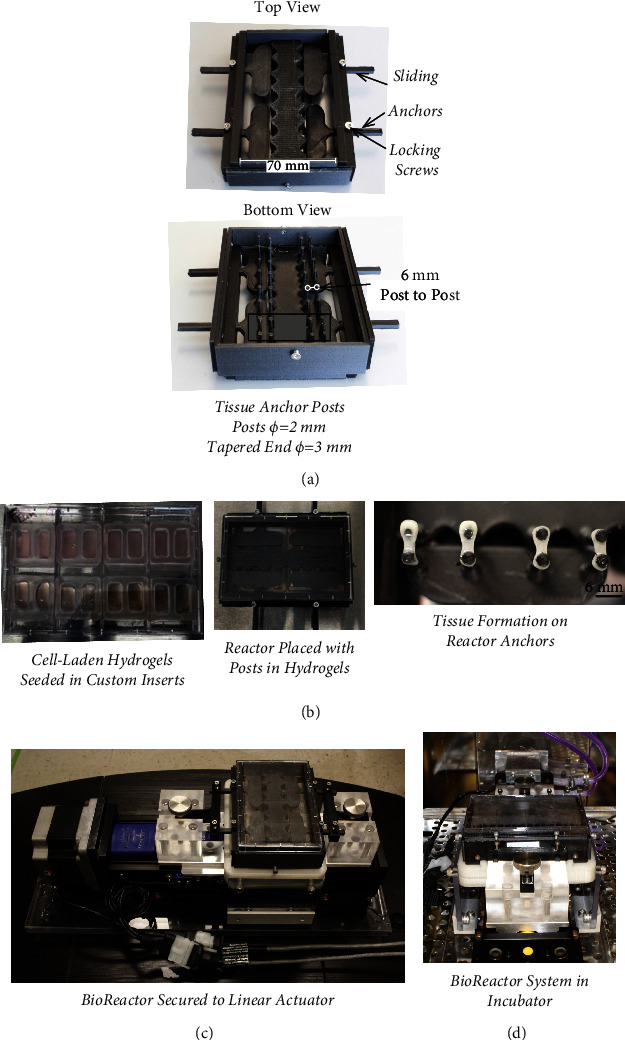
Loading of engineered tissues within the bioreactor. (a) Custom bioreactors were 3D printed with high-temperature polylactic acid. (b) Cell-laden hydrogels were seeded into custom inserts; after cells contract the hydrogels, tissues form around the anchor posts in the reactor. (c) To apply cyclic strain, the well plate is secured to a Velmex BiSlide® linear actuator with a stepper motor and a VXM controller. (d) During loading, the reactor system is placed in the incubator with a cooling plate connected to a radiator to reduce the temperature of the stepper motor by constantly circulating room-temperature fluid through the plate.

A custom, closed-system bioreactor culture housing, developed for cell-laden hydrogels and 4-well plates with custom inserts, was designed in Fusion 360 (Autodesk). The parts were fabricated as 7 separate parts with a Lulzbot Mini 2 3D printer (Lulzbot) using 1.75 mm high-temperature polylactic acid filament (Proto-pasta) (Figure 1(a)). Important features include an inner anchor as well as 4 sliding anchors, each with 4 anchor posts, 2 mm in diameter and a tapered end of 3 mm to keep tissues from coming off the ends; curvature around the posts to allow for changing media without disrupting tissues; and outer walls that secure the sliding anchors with locking screws and only allow movement in the direction of strain being applied. When anchors are in contact, the post-to-post distance, and thus, tissue length after contraction, is 6 mm. The bioreactors were made to fit on the top of a 4-well rectangular tissue culture plate (NUNC) and use the tissue culture plate's lid, allowing for a sterile environment during loading and unloading inside the bioreactor. The reactors used were assembled with stainless steel screws, and small amounts of food-safe grease were applied to the sliding anchors for the reduction of friction and to form a barrier within the reactor well plate. The reactors were sterilized by soaking for 5 min in 90% ethanol and air-drying in a biosafety cabinet under UV light for 1 hour.

Custom polydimethylsulfoxide (PDMS) inserts were fabricated with a negative mold( 3D printed with a Lulzbot Mini 2 using high-temperature PLA). Each insert had 2 wells that were 8 mm by 4 mm by 7.5 mm (L × W × H) and made to fit the width of a4-well tissue rectangular tissue culture plates, with 2 inserts per tissue culture well, allowing for 16 total tissues to be formed. PDMS culture inserts were trimmed to fit and autoclaved. Sterile inserts were placed in 4-well rectangular plates using a guide to align the wells to fit well with the bioreactor. After the inserts were aligned to the plate, they were coated in 1% BSA for 1 hr before being seeded with cell-laden hydrogels [36].

### 2.4. Seeding Cell-Laden Tendon ECM and Type I Collagen Hydrogels in the Bioreactor

Stock solutions were mixed for a final cell concentration of 3x10^6 cell/mL, and 3mg/mL for tECM or 2mg/mL for type I collagen.750 *μ*L of cell-laden hydrogel was seeded in each well for approximately 2.25 million cells per tissue (Figure 1(b)). These concentrations were the closest matching in bulk mechanical properties while still being able to form suitable tissues for culture repeatedly (Supplemental Figure 1).

After cell-laden hydrogels were seeded into PDMS inserts, sterilized culture housings were placed on the 4-well plates and anchored with screws on either side of the well plate and placed in an incubator so that the hydrogels could set. After gelation, media was added through the curvatures on the inner anchor. To cyclically load tissues, the bioreactor was mounted onto a BiSlide® linear actuator with a VXM controller (Velmex) by attaching sliding anchors to adapters on the actuator (Figure 1(c)). The linear actuator and the reactor were kept in an incubator during loading, with a cooling plate to reduce the temperature from the stepper motor (Figure 1(d)). Stimulated tissues were loaded by cycling between the seeded post distance and a verified displacement resulting in 10% strain for 10,800 cycles at 1 Hz (3 hr per day). 10% strain is a standard amount for applying mechanical stimulation in skeletal muscle constructs [30, 31, 33, 34].

### 2.5. Tissue-Level Strain Verification

Digital image correlation (DIC) was used to measure the strain of the hydrogel tissues within the bioreactor system and confirm that the prescribed displacement resulted in a 10% strain of the tissue. After tissue formation, tissues were stimulated for 3 days, with 5 min of stimulation with image capture before and after the 3-hour stimulation. Each day, hydrogel tissues were dabbed with India ink with a 10 *μ*l pipette tip in 2-3 dots along the length of the tissue, to increase contrast within images of the tissues. The linear actuator was outfitted with camera towers to allow for displacement cycles to be applied by using the VMX BiSlide system, with visualization under the plate of the tissues for stimulation with image capture. Images were captured with an iPhone 12 back camera at 4K resolution and 30 fps (Supplemental Figure 2).

10 cycle subsets of stimulation with image capture were used for further analysis. 10 cycle videos were rendered into individual images in Adobe Photoshop. Regions of interest were defined by masking individual tissues in Photoshop. The images were then uploaded into open source, 2-D DIC MATLAB software, nCorr [37]. DIC backward analysis was performed with a subset radius of 30 pixels, spaced apart in increments of 5, to map displacements between the original state and the tissue before and after each cycle. Lagrangian strain with a strain radius of 15 was used to calculate a strain field over the tissue. The average strain for each tissue was quantified at peak displacement for each cycle. To evaluate strain over 3 days, the average strain at peak displacement for each was averaged for the individual tissues.

### 2.6. Static and Stimulated Culture to Evaluate MTJ Gene and Protein Expression

**Figure 2:**
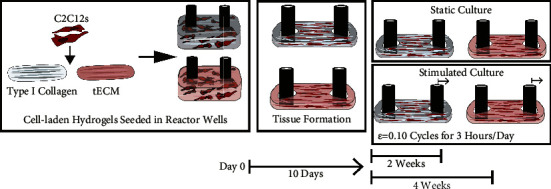
Experimental methods to study the effects of mechanically stimulating C2C12 and ECM hydrogel tissue constructs. Briefly, C2C12 myoblast cells were seeded in either type I collagen or tECM hydrogels; then, the cell-laden hydrogel was transferred to a reactor well. Within the culture wells, the cells contracted the hydrogel-forming tissue around the reactor posts after 10 days. Tissues were either cultured in a static condition with no cyclic strain loading or loaded to a strain of 0.10 cyclically for 3 hr per day. Tissues were cultured for 2 and 4 weeks before endpoint analysis to study gene and protein expression.

Engineered tissues were cultured over 3–5 weeks to evaluate MTJ gene and protein expression. Cell-laden hydrogels were seeded and cultured for 10 days to allow for complete tissue formation and attachment to the tissue anchors before any loading cycles. After tissue formation, the tissues were cultured for 14 and 28 days in either static or stimulated culture conditions (Figure 2). Static tissues were cultured alongside the stimulated tissues in the same plate configurations with no loading applied. At the respective endpoints, the tissues were harvested for immunohistochemistry and qPCR (Figure 2).

### 2.7. Gene Expression of Muscle and Muscle-Tendon Junction Markers

At the conclusion of 2 weeks or 4 weeks of the culture, 3 tissues for each condition were extracted from the reactor. The tissues were homogenized with 1.75 mm ceramic beads in a Beadmill 24 tissue homogenizer (Fisher Scientific). After homogenization, RNA was isolated with the EZNA RNA isolation kit (Omega Bio-tek). qPCR was performed using a QuantStudio 3 PCR machine (Thermo Fisher) and SYBR Select Master mix (Thermo Fisher). Primers for genes of interest can be found in Supplemental Table 1. For *Pax* and *Col22a1*, three samples from each condition were run separately with technical replicates. Samples were pooled for biological averages for tECM at 2 weeks and 4 weeks and type I collagen at 2 weeks to evaluate myogenic differentiation genes, *Dys*, *Myh1*, *Myh2*, and *Myh4*. Fold changes of ∆CT values normalized to GAPDH were used for quantification.

### 2.8. Immunohistochemistry of Paxillin and Type XXII Collagen

After the culture, the tissues were fixed in 4% formaldehyde for 15 min, washed with PBS, and then soaked in 10%, 20%, and 30% sucrose solutions for ten minutes each. The tissues were embedded in OCT and frozen at −80°C. The tissues were cryosectioned, in plane with the axis of loading, in 10 um, and slides were stored at −20°C before staining. The samples were rinsed in ice-cold PBS for 10 min, permeabilized in 0.5% triton-X 100 for 15 min, and rinsed in cold PBS for 10 min. The samples were blocked in 1% BSA overnight at 4°C. The samples were incubated at 37°C for 2 hr in a rabbit anti-paxillin antibody (Millipore-Sigma), which was used at 1 : 100 dilution in 1%BSA or rabbit anti-COL22A1 antibody (Thermo Fisher) at 1 : 200 dilution in 1%BSA. Anti-Rabbit Donkey AlexaFluor 594 (Invitrogen) was applied for 30 min at room temperature. The samples were rinsed, stained with DAPI for 5 min at room temperature, and sealed with a Prolong Gold Antifade mountant (Thermo Fisher). Secondary-only controls for representative images are shown in Supplementary Figure 52.

For image analysis, 3 slides per sample, taken from the top, middle, and bottom, were used to control for variation within the samples. Representative images were selected, and all the images were analyzed quantitatively. There were no qualitative or quantitative differences between the depth of the sections (Supplemental Table 2). Thresholds were applied to individual channels in ImageJ to isolate the area of the positive signal for each channel. The ratio of TXRed and DAPI positive areas was defined as the Expression Index (E.I.)

### 2.9. Statistical Analysis

All statistical analysis was performed with GraphPad Prism (v 9.4.1). For statistical comparison of gene expression values, *n* = 3 tissue samples were used as biological replicates (averaged technical replicate of *n* = 2). Student's *t*-tests were used to compare the ∆CT values for each material in either the static or stimulated culture groups. For statistical comparison of E.I. values to quantify protein expression, *n* = 3 tissues were measured in 6 areas (3 separated sections, 2 areas per section). Those measurements were averaged for a single E.I. for each tissue sample. Student's *t*-tests were used to compare the E.I. values for each material in either the static or stimulated culture groups.

## 3. Results

### 3.1. Digital Image Correlation for Tissue Strain Analysis Is Controllable with a Hydrogel Bioreactor

**Figure 3:**
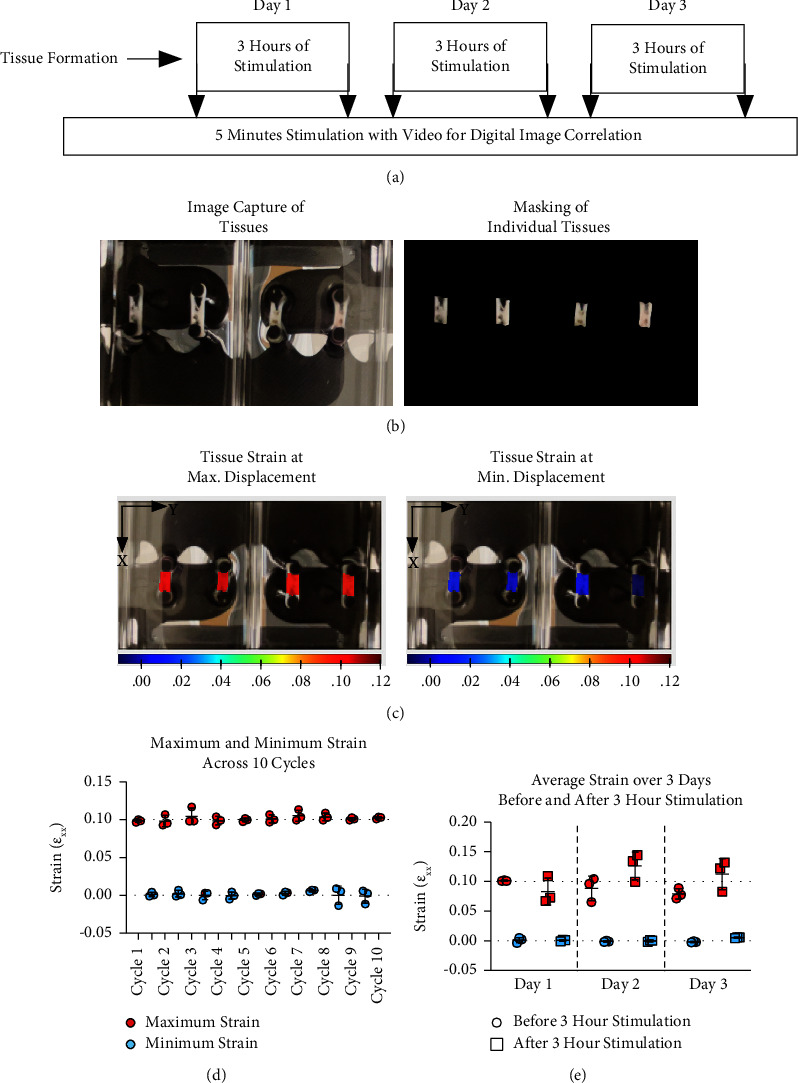
To verify the calculated displacement resulted in the expected strain, tissues were analyzed with DIC to determine strain across 3 days of mechanical stimulation. (a) Over three days, tissues were observed for 5 min before and after 3 hr of mechanical stimulation. (b) Videos acquired were rendered into individual images for DIC, and the region of interest was defined in Photoshop by masking individual tissues without the posts. (c) After DIC analysis, tissues had uniform strain throughout at peak and minimum displacement around the target values. (d) After tissue formation (day 10) and before any 3-hour mechanical stimulation, 10 loading cycles resulted in strains averaging 0.10 at maximum displacement and 0.01 at minimum displacement. (e) Over 3 days of observation, before and after 3 hours of loading, strains at maximum displacement averaged 0.10 and 0.00 at minimum displacement.

The strain of engineered tissues was measured 6 separate times over 3 days by recording 5 min of loading before and after 3 hr of cyclic loading with a modified reactor system (Figure 3(a)). After image capture and selection of regions of interest (Figure 3(b)), heatmaps of the individual tissues at minimum and maximum displacements indicate that strain is consistent throughout the tissue (Figure 3(c)). Prior to long-term loading, quantification of the strain over 10 cycles before a 3-hour loading cycle in each of the 3 tissues was 0.10 ± 0.01 at maximum displacement and 0.00 ± 0.01 at minimum displacement, indicating the ability of the system to apply consistent strain. Overall, 0.10 strain was achieved in the first observation, after which average strain loading slightly deviated from the target strain, with very low standard deviations across measured cycles. Over 3 days before and after, strain in the tissues at maximum displacement was 0.09 ± 0.02 with a range of 0.08 and 0.11, both occurring on day 3. In addition, all tissues had a similar average strain at the maximum displacement and returned to near zero strain after each cycle (average strains of 0.01 ± 0.01 with a range of −0.01 and 0.01). In all cases of the measured maximum strain, the standard deviation of the ten cycles each day was less than 0.02; at minimum strain, the standard deviation among cycles was 0.01.

### 3.2. Gene Expression of Paxillin and Type XXII Collagen Is Affected by Mechanical Stimulation and Hydrogel Constituents

**Figure 4:**
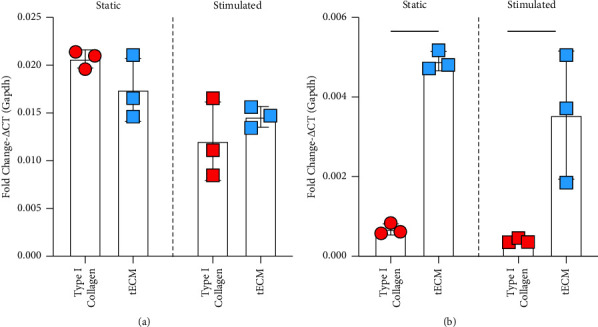
Relative gene expression determined with qPCR of *Pax* and *Col22a1*. (a) Expression of *Pax* was not significantly different in different hydrogel materials in static and stimulated cultures. (b) *Col22a1* gene expression was markedly upregulated in tECM hydrogels compared to type I collagen (^*∗∗∗∗*^indicates *p* < 0.0001; ^*∗*^indicates *p* < 0.05).

For *Pax* expression, statistically significant differences in relative expression were not identified between type I collagen and tECM (Figure 4(a)). ECM material significantly affected *Col22a1* expression in static and stimulated culture conditions, with 6- and 10-times higher expression in static and stimulated cultures, respectively (*p* < 0.01, Figure 4(b)). In addition, constructs were evaluated to observe gene expression patterns that would suggest myoblast differentiation (Supplemental Figure 3). Myosin heavy chains 1, 2, and 4 (*Myh1*, *Myh2*, and *Myh4*) were upregulated after loading in tECM; however, these were downregulated after loading in type I collagen tissues. *Dys* was upregulated in tECM tissues, compared to type I collagen at 2 weeks, and expression increased in tECM at 4 weeks. With comparisons between hydrogels and culture conditions with two-way ANOVA, it was observed that with respect to *Pax* expression, there was a significant difference between static and stimulated cultures (*p* < 0.01) but not between hydrogels. For *Col22a1*, there were significant differences between hydrogels, but not in static or stimulated culture conditions (*p* < 0.0001). There was no interaction effect for both genes of interest.

### 3.3. Paxillin Protein Expression Is Increased in tECM Tissues, and Mechanical Stimulation has an Additive Effect after 4 Weeks of the Culture

**Figure 5:**
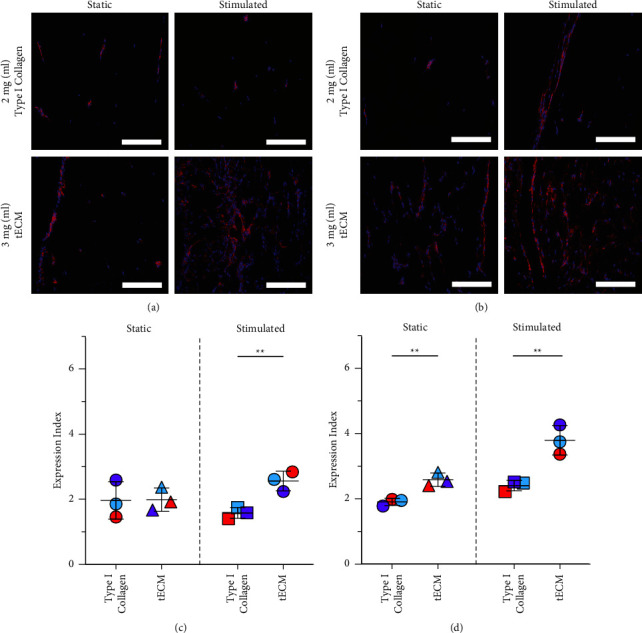
Paxillin protein expression was more evident in tECM tissues and highest in tECM tissues stimulated during the culture. (a, b) Representative images of samples stained with antipaxillin (red) and DAPI (blue) at 2 and 4 weeks, and scale bars are 200 *μ*m. At both time points, staining is more evident in tECM tissues, specifically those with loading. (c, d) E.I. values were calculated by normalizing the positive area of the antipaxillin fluorescent channel, TXRED, and the DAPI fluorescent channel. Student's *t*-tests were used to compare the hydrogel materials in static or stimulated conditions. Large differences in paxillin expression are not seen after 2 weeks of the static culture; in the stimulated culture conditions, there was a significant difference between hydrogel materials (^*∗∗*^indicates *p* < 0.01). After 4 weeks, statistical differences between materials in both the culture conditions were observed.

Protein expression was evaluated with immunohistochemistry, and the area of positive pixels was used to quantify the expression in the varying conditions. After 2 weeks of the culture, increased staining and cellularity in the 3 mg/mL tECM hydrogel groups were clear compared to those in 2 mg/mL type I collagen (Figure 5(a)). While differences were not apparent between static and stimulated groups of type I collagen hydrogels, there was high expression throughout the stimulated tissues of tECM compared to the static group. After 4 weeks of the stimulated or static culture, increased protein expression was more apparent in the stimulated groups than in the static groups cultured in the same hydrogel materials (Figure 5(b)). The highest expression still occured in tECM, and in the stimulated group, staining was evident throughout the entire tissue. At 2 weeks, there was no significant effect of hydrogel materials or culture conditions, determined with two-way ANOVA. However, at 4 weeks, hydrogels and culture conditions both had significant effects on the relative expression of paxillin (*p* < 0.001). There were no interaction effects.

Once normalized to cellularity, quantitative measures of protein expression (E.I. values) were similar to qualitative observations. At 2 weeks, differences between the two materials in the static culture condition were minor (1% change). In the stimulated culture conditions, there was a significant difference between the two hydrogel materials (*p* < 0.01), with a 62% increase (Figure 5(c)). After 4 weeks of the culture, there were statistically significant differences between hydrogel materials in both culture conditions (*p* < 0.01) (Figure 5(d)). There was a more significant difference between materials in the stimulated culture conditions compared to the difference in materials in the static culture conditions (a 57% increase vs. a 31% increase, respectively). The analysis of the positive area of individual channels indicated that there were more significant differences in the groups in paxillin expression, quantified by TXRED, as well as larger increases in cellularity, quantified by DAPI (Supplemental Figure 4).

### 3.4. Type XXII Collagen Protein Expression Is Increased in tECM Tissues and Is Greater at 4 weeks of Culture in Mechanically Stimulated Conditions

**Figure 6:**
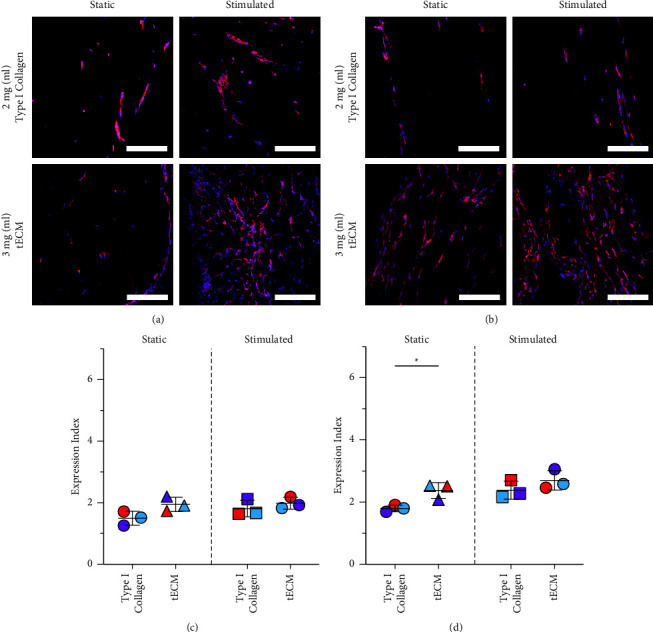
Type XXII collagen expression was more evident in tECM tissues and highest in tECM tissues stimulated during the culture. (a, b) Representative images of samples stained with anti-Col22a1 (red) and DAPI (blue) at 2 and 4 weeks, and scale bars are 200 um. In each, staining is more evident in tECM tissues and is more prevalent throughout the tissues in stimulated tECM tissues. (c, d) E.I. values were calculated by normalizing the positive area of the anti-Col22a1 fluorescent channel, TXRED, and the DAPI fluorescent channel. After 2 weeks of the culture, there were no measurable differences in hydrogel materials in the static or stimulated culture as determined with Student's *t*-tests. After 4 weeks of the culture, more significant differences in type the XXII collagen Expression Index in different materials are evident in the static culture (^*∗*^indicates *p* < 0.05). Stimulated culture conditions did not have significant differences between hydrogel materials.

Type XXII collagen protein expression had trends similar to those of paxillin expression. After 2 weeks, expression was evident in all tissues and appeared to be more abundant in stimulated tissues than in nonstimulated tissues for each hydrogel material (Figure 6(a)). Stimulated tECM tissues seemed to have the highest cellularity of all the culture conditions and, like the results for paxillin, had the highest protein expression throughout the tissue sample. After 4 weeks, cells in tECM cultured in static conditions also began showing increased expression compared to type I collagen. Both the stimulated groups maintained higher levels of expression than their static counterparts (Figure 6(b)).

Interestingly, quantification of the normalized expression of type XXII collagen at 2 weeks showed little to no difference between groups (Figure 6(c)). After 4  weeks of the static or stimulated culture, the increase observed in qualitative images was evident, especially in static tECM tissues, with a 32% increase in E.I. in tECM compared to type I collagen (*p* < 0.05) (Figure 6(d)). In comparisons with two-way ANOVA, we observed a significant effect of the hydrogels on type XXII collagen protein expression at two weeks and significant effects from hydrogels and culture conditions at 4 weeks (*p* < 0.05).

## 4. Discussion

Studying mechanical stimulation and its effects on signaling and protein expression in cells around the MTJ is important when understanding homeostasis and the junction's developmental process. This information will help incorporate multiple tissue-specific materials and cell types in future models that can be used to test new therapies to improve healing. A custom bioreactor allowed for mechanical stimulation of C2C12 myoblast cells in tendon ECM hydrogels, which previous research has shown to promote MTJ-like protein expression. After 2 and 4 weeks of cyclic loading of a 10% strain for 3 hr a day, protein expression of paxillin and type XXII collagen protein expression was upregulated. Overall, this system took the next steps toward applying mechanical stimulation to cell constructs that mimic the MTJ *in vitro.*

Applying physiologically relevant mechanical strain to cells in culture is key when studying *in vitro* tissue systems, especially in the musculoskeletal system. We successfully designed a bioreactor based on a traditional well plate that can be combined with tissue-specific hydrogels to study the effects of specific mechanical stimuli and hydrogel types on MTJ protein expression. Although we cannot recreate the entire set of stimuli present during MTJ development, the novel bioreactor described here offered three advantages over other bioreactor systems: the reactor housing is easily manufactured with a 3D printer using inexpensive materials (high-temperature PLA); it is contained entirely within a commercial well plate and thus can be used for stimulated constructs much like in a standard cell culture plate, and finally, it allows for the use of tissue-specific hydrogel biomaterials to make engineered tissue constructs. Tendon extracellular matrix hydrogels have been previously shown to increase the expression of MTJ-related genes [15]. Based on this previous study, we selected tendon ECM combined with mouse myoblasts to understand the role that mechanical stimuli play in muscle-tendon junction biology in a relevant microenvironment.

Strain applied at the tissue level is important when applying mechanical stimulation. A 10% strain was selected due to its prevalent use in previous studies using C2C12 myoblasts [30, 31, 33, 34]. The tissue-level strain was measured over 3 days, before and after 3 hr of mechanical stimulation. As expected, the displacement resulted in a strain average of 0.10 ± 0.01 over 10 cycles before 3 hr of mechanical stimulation. This indicates that the bioreactor system applies the intended strain before major bouts of loading. Discrepancies in tissue strain among cycles can be explained by out-of-plane strains being a factor resulting from the alignment or disalignment of the tissues. These deviations from a 10% strain were less on day 3 than those on day 2, suggesting that there may be some convergence over time due to tissue alignment, which would be an interesting future assessment. These data supported that the hydrogel bioreactor could consistently apply the intended strain over several days of the stimulated culture conditions.

Our main research focus was the detection of MTJ-specific proteins such as paxillin and type XXII collagen to determine if mechanical stimulation increased paxillin expression and promoted type XXII collagen synthesis in C2C12 myoblasts. When measuring the gene expression of these two proteins after two weeks of the culture, *Pax* expression was highest in type I collagen tissues in the static culture. Gene expression was lower for both hydrogels after 2 weeks of the mechanical stimulation culture. Interestingly, Col22a1 gene expression was upregulated in both static and stimulated culture conditions of tECM tissues, with the highest expression in the static condition group. As gene expression is a snapshot of the cell's response, it is hard to say whether this trend stays consistent or if this expression pattern varies.

Similar to the previous work, tECM influenced paxillin protein expression more than type I collagen hydrogels [15]. In combination with mechanically stimulated cultures, expression increased in tECM compared to type I collagen, and the increase in expression was more than two times higher in the stimulated culture conditions. This increase suggested that the myoblast cells responded to mechanical stimulation by forming more adhesions. These results indicate that tECM supported those pathways more than type I collagen. This would be expected as paxillin expression increases due to mechanical loading during development [23, 24]. Both materials had upregulation of type XXII collagen after 4 weeks of the mechanically stimulated culture. This was expected as extracellular matrix protein expression would rise in response to mechanical stimulation [8]. Our data suggest that mechanical stimulation had the largest effect on protein expression. It is also evident that more extended culture periods are needed to analyze type XII collagen protein expression, as the most significant differences between expression occurred after 4 weeks of the stimulated or static culture. This supports other studies that observed that type XXII collagen is expressed by muscle cells at the MTJ [6–8]. This is interesting as there is still debate whether connective tissue cells such as tendon fibroblasts contribute to type XXII collagen production or if it is primarily produced by muscle cells.

Although the present study provides important information that helps explain the expression of MTJ-specific proteins, the current approach has limitations. The myoblasts used in the current study are useful in understanding the interaction between the specialized cell niche and the tendon matrix. As mentioned previously, studies using a 10% strain showed improved myogenic differentiation [30, 31, 33, 34]. Our constructs were evaluated for gene expression of myosin heavy chain genes (*Myh1*, *Myh2*, and *Myh4*), a good indicator of muscle fiber formation, as well as dystrophin (*Dys*), cell adhesion in muscle cells in muscle tissue (Supplemental Figure 3) [3, 38]. Evaluation of gene expression of these markers did not indicate myogenic differentiation of C2C12s in our system. Compared to other approaches looking at myoblasts in mechanically stimulated conditions, this study did not result in myoblast fusion, as evidenced in IHC staining or myogenic differentiation determined by gene expression. Primary myoblasts could offer several benefits that immortalized C2C12s cannot, such as increased expression of muscle-specific markers of differentiation (*Myod* and *Myog*) and more mature muscle constructs [39, 40]. It should be considered for future studies to include differentiated muscle cells as part of this model or to optimize the tissue construct to promote myogenic differentiation.

Cyclic strain application was verified over several days and observed to be consistent day to day, with a target strain of 10%. Limitations were evident in the observation in analysis because of low-resolution imaging. Here, we used lower resolution image correlation parameters to reduce noise measured within the tissue. Further verification of the true localized strain levels would be useful and allow for different amounts of strain to further elucidate the cell's responses to mechanical signaling. Nevertheless, for this study, there was constant and measurable strain within the tissues, similar to what we expected from applied mechanical loading, which was consistent across tissues within each reactor.

This study compared stimulated tissues to those cultured in a static condition. However, there is still some form of mechanical signaling that arises from the tissue contraction around the rigid posts. While there were some differences between groups, it is hard to completely remove mechanical signaling from the system without using inhibitors. Bulk mechanical properties of the hydrogels were evaluated, with tECM hydrogels having a higher complex modulus than type I collagen hydrogels. After water expulsion from the gel, these properties likely changed drastically. Evaluating the stiffness of the constructs after contracture, as well as after mechanical stimulation, is needed to assess whether mechanical properties of the engineered constructs approach native tissue.

*In vitro* systems, like the one presented, take a significant step toward modeling the muscle-tendon unit, which can be used to study the development and disease of the muscle and tendon tissue. Here, the MTJ is modeled with muscle cells and a tendon environment in a system that produces expected responses *in vivo*; i.e., paxillin expression increased in response to mechanical loading. With regard to junction-specific biology, a system like this could be utilized to determine pathways that could mitigate negative outcomes because of disease, such as muscular dystrophy, at the MTJ. The system could also be used in conjunction with *in vitro* muscle-tendon units to study inflammation resulting from chronic overuse by varying the mechanical loading environment. In addition, this system supports the need to incorporate not only muscle and tendon tissue in a model but also a relevant connection between the tissues.

The described system has several advantages over the current methods, making it an attractive platform to study the muscle-tendon unit, specifically the MTJ. The novel well plate bioreactor can be used to study mechanical stimulation of engineered tissue constructs using ECM hydrogels and is a system that could be applied to many different engineered tissues using hydrogels. The tECM constructs could be stimulated for multiple weeks to determine the effect of mechanical stimulation on cells at the myotendinous junction. Future studies should include outcome measures to assess functional differences of these tissue constructs, such as mechanical testing of the tissues or matrix production studies. These, along with protein expression, may better inform regenerative medicine strategies to address the need for *in vitro *muscle-tendon units.

## Data Availability

The data that support the findings of this study are available from the corresponding author upon request.

## Supplementary Materials

Supplementary Materials A file, “Supplemental Figures and Tables,” is included with this manuscript. This file contains tables and figures that detail prime information for gene expression, gene expression analysis for myogenic differentiation markers, methods and results for rheological properties of engineered tissues, modifications to the bioreactor system to capture images during strain cycles, and data from individual fluorescent channels for immunohistochemistry. In addition, STL files as well as detailed instructions are included for assembly of the bioreactor housing.
