# Supplementary material for: Tendon Extracellular Matrix Promotes Myotendinous Junction Protein Expression in Engineered Muscle Tissue under Both Static and Mechanically Stimulated Culture Conditions
Source: J Tissue Eng Regen Med. 2023 Aug 29;2023:6658543. doi: 10.1155/2023/6658543 (PMC11918950; doi:10.1155/2023/6658543)
Supplement: Supplementary Materials — A file, “Supplemental Figures and Tables,” is included with this manuscript. This file contains tables and figures that detail prime information for gene expression, gene expression analysis for myogenic differentiation markers, methods and results for rheological properties of engineered tissues, modifications to the bioreactor system to capture images during strain cycles, and data from individual fluorescent channels for immunohistochemistry. In addition, STL files as well as detailed instructions are included for assembly of the bioreactor housing. [file 6658543.f1.docx]

Supplemental Materials:

Supplemental Table 1: Primer sequences for primers used for gene expression analysis. All species are mus musculus. Forward (FR) and reverse (RV) sequences are presented.

**Supplemental Table 2:** p-values determined with one-way ANOVA with repeated measures to determine differences between differences from the depth of tissue sectioned. No significant differences were observed between tissue depths.

Supplemental Figure 1: A) Rheological properties of the hydrogel materials influence the ability of cells to contract the tissue around the reactor posts. B) Viscosity was measured in two concentrations of type I collagen, 2 mg/mL and 0.5 mg/mL and 3 mg/mL tECM. The lower concentration of type I collagen had similar viscosity values across the frequency sweep as the tECM, while the higher concentration of type I collagen had much higher values. C) Engineered tissues had different amounts of contraction, eight tissues per group were outlined to measure area of the tissue, which was normalized to the area of the well. As seen qualitatively, tECM contracted 85% of its original area after only 24 hours. While 2 mg/mL type I collagen took much longer to contract and had contraction of 25% of its area at the time of seeding. After 10 days, type I collagen tissues contracted to 30% of the original area.

**Supplemental Figure 2:** To visualize tissues during cyclic displacement, camera towers were used to lift the well plate on the linear actuator. Then a camera was fitted under the plate to capture video during the displacement cycles. The well plate was anchored with a clamp and ring stand to prevent movement.

Supplemental Figure 3: Muscle specific gene expression in static and stimulated tissues for 2mg/mL type I collagen and 3mg/mL tECM tissues cultured in the bioreactor system for two weeks and 3mg/mL tECM tissues cultured in the bioreactor system for four weeks. Dystrophin was most up-regulated in tECM after culture for 4 weeks, and that up-regulation was not as much in the stimulated tissues. Type I collagen groups had up regulated MHC genes compared to tECM especially in non-stimulated groups. In the stimulated groups tECM upregulated the myosin heavy chain genes more, especially at 4 weeks.

Supplemental Figure 4: Areas of positive pixels in each channel, DAPI for cell nuclei and TXRED for paxillin or type XXII collagen, for tissues after 2 weeks and 4 weeks of culture in the bioreactor culture system. These values are used in the expression index for the ratio of target protein stain to the cell nuclei present in the sample. Consistently, the target protein is most up regulated in the stimulated tECM groups. DAPI positive area is also increased in the tECM tissues compared to type I collagen tissues. In these comparisons, Dapi area in the analysis was consistently increased in stimulated tECM cultures at both 2 weeks and 4 weeks. At 4 weeks of culture, Dapi area was significantly higher in tECM cultures than in any condition of type I collagen cultures. With respect to the proteins of interest, at 2 weeks, the only significant increase in area was in stimulated cultures of tECM. At 4 weeks, both conditions of type I collagen were significantly lower than static tECM cultures, which was significantly lower than stimulated tECM cultures.

Supplemental Figure 5: Representative samples, used in figure 5 (A and B) and 6 (C and D), stained with the same staining protocol, without any primary antibody. With only the secondary antibody, we have verified that there is no non-specific binding of the secondary antibody.

## **Supplemental Methods:**

## **Rheological Properties of ECM hydrogels and resulting tissue contraction**

Bulk mechanical properties of the ECM hydrogels were measured with rheology using an MCR 92 (AntonPaar, Graz, Austria) and following the parameters established by Freytes *et al*.[28] Oscillatory shear strain of five percent strain was applied over a frequency of .5 rad/s- 100 rad/s, then from 100 rad/s to .5 rad/s with 5 measurements per decade. Briefly, 3mg/mL hydrogels from tECM, and 2 mg/mL hydrogel of type I collagen were seeded onto the rheometer after neutralization and buffering. The 25 mm parallel measuring plate (Anton Paar, Graz, Austria) was lowered onto the solution left for 45 min at 37C to allow for full self-assembly of the hydrogel before initiating the frequency sweep. *N* = 3 samples from each material were tested. Data are presented as the mean, with the standard deviation shaded on a logarithmic scale (Figure 2A). Images of tissues in the bioreactor systems were taken at 24 and 72 hours and analyzed in ImageJ to determine percent contraction of the hydrogels.
